# Neurobiological influence of comorbid conditions in young patients diagnosed with gaming disorder: A whole-brain functional connectivity study based on a data driven method

**DOI:** 10.1371/journal.pone.0233780

**Published:** 2020-05-29

**Authors:** Shinya Kuriki, Susumu Higuchi, Hideki Nakayama, Satoko Mihara, Yasuomi Okazaki, Yumie Ono, Hiroshi Kobayashi

**Affiliations:** 1 School of Science and Engineering, Tokyo Denki University, Saitama, Japan; 2 Faculty of Health Sciences, Hokkaido University, Sapporo, Japan; 3 National Hospital Organization Kurihama Medical and Addiction Center, Yokosuka, Japan; 4 Advanced Science and Engineering, Waseda University, Tokyo, Japan; 5 School of Science and Technology, Meiji University, Kanagawa, Japan; 6 School of Information Environment, Tokyo Denki University, Tokyo, Japan; Tokyo Metropolitan Institute of Medical Science, JAPAN

## Abstract

Gaming disorder, which is characterized by multiple cognitive and behavioral symptoms, often has comorbid psychiatric conditions such as depression and attention-deficit hyperactivity disorder. Neurobiological effects of the comorbid disorders so far reported are not converging, exhibiting positive and negative alterations of the connectivity in brain networks. In this study, we conducted resting-state functional magnetic-resonance imaging and whole brain functional connectivity analyses for young participants consisting of 40 patients diagnosed with the gaming disorder, with and without comorbid conditions, and 29 healthy controls. Compared to healthy controls, the gaming disorder-alone patients had partially diminished connectivities in the reward system and executive control network, within which there existed central nodes that served as a hub of diminished connections. In the gaming disorder patients who had comorbidity of autism spectrum disorder, the diminished connections were enlarged, with alteration of the hub nodes, to the entire brain areas involved in the reward system including cortical, subcortical and limbic areas that are crucial for reward processing, and to the whole cortical areas composing the executive control network. These observations suggest that the neurodevelopmental condition coexisting with the gaming disorder induced substantial impairment of the neural organizations associated with executive/cognitive and emotional functions, which are plausibly causal to the behavioral addiction, by rearranging and diminishing functional connectivities in the network.

## Introduction

Gaming disorder has been defined in the International Classification of Diseases, the Eleventh Revision for Mortality and Morbidity Statistics, World Health Organization (ICD-11) [1], as a pattern of gaming behavior characterized by impaired control over gaming, increasing priority given to gaming over other activities, and continuation or escalation of gaming despite the occurrence of negative consequences. Previous studies on clinical and behavioral features of the Internet gaming disorder (IGD), a gaming disorder associated with Internet use (more generally, Internet addiction: IA), have revealed coexistence of psychiatric disorders with the IGD. Of these, the most commonly observed disorders include depression and attention-deficit hyperactivity disorder (ADHD) [2–6]. A meta-analysis reported significantly higher proportion of patients with depression in individuals with IA than in healthy controls [7]. A review article [8] also described that the prevalence of ADHD was higher in IA subjects than in non-IA subjects. The overall severity of ADHD symptoms in IA groups was significantly worse than in healthy control. Diverse comorbid psychological pathologies were reported to have association with IGD; 92% of reviewed studies had significant correlations with anxiety, 89% with depression, 87% with ADHD or hyperactivity symptoms, and 75% with social phobia/anxiety and obsessive-compulsive symptoms [9]. Meanwhile, measures of depression and autistic traits had strong correlation with scores on Internet Addiction Test in a group consisting of about half-and-half lower and higher problematic Internet-use groups [10]. Psychiatric patients who were problematic Internet users had significantly higher scores than normal Internet users on self-rated scales of sleep problems, depression, trait anxiety, ADHD, autism, obsessive-compulsive disorder, social anxiety disorder, and impulsivity [11]. The last two studies imply that the developmental disorder of autism spectrum disorder (ASD) has close associations with IGD as well as ADHD. Thus, the comorbidity of ASD may be expected to impact the symptoms of IGD patients.

In a cellular and molecular model of drug dependence, the region of nucleus accumbens in the ventral striatum, which is a target of dopaminergic links from the ventral tegmental area in the midbrain, is the central structure receiving projections from the olfactory cortex, amygdala and limbic cortex [12]. The mesolimbic circuit including the nucleus accumbens, amygdala and hippocampus was hypothesized to be the key zone that mediates the rewarding and reinforcing effects related to drug intake [13]. The dopamine innervation in the mesolimbic circuit extends to the anterior cingulate cortex and orbitofrontal cortex in the forebrain to form the mesocortical dopamine system. The orbitofrontal cortex and anterior cingulate cortex were most frequently implicated in drug addiction to be activated during intoxication, craving, and bingeing but deactivated during withdrawal [14]. These cortical regions were proposed to be involved in cognitive and emotional functions and supposed to regulate motivation, drive, and self-control [15].

As for the behavioral addiction, an fMRI activation study for pathological gambling individuals using Stroop task showed diminished response in the ventromedial prefrontal cortex, which was implicated in poor impulse control [16]. In other fMRI study, reduction of activation in the ventral striatum and ventromedial prefrontal cortex was observed in pathological gamblers during a guessing task that was known to otherwise activate the ventral striatum. The magnitude of activation was negatively correlated with gambling severity, implying a deficiency of the mesolimbic reward system [17]. Problematic gamblers and heavy smokers commonly showed reduced responsiveness of the dorsomedial prefrontal cortex, compared to healthy controls, during successful as well as failed response-inhibition in a stop signal task [18]. The cortico-striatal neural circuit comprising the orbitofrontal cortex, ventromedial prefrontal cortex, anterior cingulate cortex and the striatum was implicated in reward responsiveness, impulsivity and compulsivity in the impulse control disorders that are conceptualized as the behavioral addiction [19]. The mesolimbic and mesocortical reward systems were suggested as the central brain mechanisms underlying the behavioral addiction in congruence with drug/substance-use addictions [20].

In IGD/IA subjects compared to controls, alteration of different variables associated with striatal activities was reported. Those activities include: reduced dopamine expression level of the striatum [21]; reduced levels of dopamine D2 receptor availability in subdivisions of the striatum including the bilateral dorsal caudate and right putamen [22]; decreased fMRI activation in the ventral striatum during the anticipation of monetary rewards [23]; reduced functional connectivity of the dorsal putamen with posterior insula-parietal operculum [24]; reduced activation in the striatum, inferior frontal gyrus and presupplementary motor area during Go-Stop task [25]; and reduced functional connectivity of the prefrontal cortex with dorsal striatum, pallidum and thalamus which are involved in the prefrontal-striatal circuit [26]. The outcomes of these studies imply depressed activities of the striatum-related structures in the IGD and cognitive conditions. Based on these findings, observations of wide brain regions, including the prefrontal cortex, limbic structures and other dopamine-innervated areas are desirable to clarify the impact of IGD on the brain activities.

A functional connectivity study with resting-state (rs-) fMRI is a versatile neurobiological approach requiring no tasks during recording; the procedure is suited for clinical use especially for young patients, who may have difficulty in responding appropriately to activation tasks. Prior functional connectivity studies on IGD and IA predominantly employed a seed-based approach using brain regions which were determined based on a priori knowledge, models, or hypotheses [23, 25, 27–43]. Key regions in the reward circuit related to addiction, such as the striatum, nucleus accumbens, orbitofrontal cortex, anterior cingulate cortex and insula, have frequently been used as the seeds. Diminished connectivities of these seeds with other brain areas were broadly observed in IGD/IA subjects compared to controls, while enhancement of the connectivity was also found.

As for the influence of major comorbid conditions of ADHD and depression on the IGD, several studies have reported alterations of rs-functional connectivity. IGD males with comorbid depression had diminished connectivity of a seed in sub-areas of the anterior cingulate cortex with the prefrontal cortex, compared with IGD males without depression, while the connectivity of the same seed was enhanced with the precuneus and posterior cingulate cortex [41]. Negative mood states of depression and anger reflected poorer or impaired connectivity among the default mode network (DMN) regions in IGD males, compared to healthy controls [36]. Posterior cingulate cortex-seeded connectivity was expanded in IGD subjects without childhood ADHD, compared to those with childhood ADHD, in the brain regions implicated in salience processing [38]. Within IGD subjects, depression scores were negatively correlated with the connectivity between the left amygdala and right dorsolateral prefrontal cortex [42]. IGD subjects with major depression disorder and/or ADHD had enhanced connectivity of DMN and executive control network (ECN) compared to healthy controls [37]. The results of these seed-based functional connectivity studies are not convergent; comorbidity of ADHD and depression either enhanced or reduced the connectivities in brain regions related to different networks.

Although the seed-based approach is effective to identify altered connections with the region of interest, it cannot provide a comprehensive picture of the whole brain because the number of seed regions is limited. To explore large scale functional organization, network-based statistics have been used to examine functional connectivity in the entire brain. The underlying principle of the network-based statistics relies on the interconnectedness of node links in topological space [44], which tends to yield long significant links going through multiple nodes. Several studies based on the network-based statistics have reported alterations of the connectivity links in IGD/IA individuals compared to controls. Those altered connectivity links included: cortico-subcortical circuits with reduced values of functional connectivity, in which the putamen was most extensively involved [45]; disrupted long-range connections in the frontal, parietal, and occipital lobes having negative and positive functional-connectivity differences between IA and healthy subjects [46]; and the networks interconnected mostly in frontal regions where functional connectivity was positively- and negatively-correlated with the score of Internet Addiction Test in healthy subjects [47]. The whole brain network-based statistics approach permits identification of altered long connections through many nodes. However, it is difficult to disentangle the identified multiple connections, which are usually overlapped, into known functional networks or reward circuits.

In this study, we aimed to evaluate the influence of comorbid conditions on the direction and extent of the alteration of functional networks in individuals diagnosed with gaming disorder (GD) based on the criteria in ICD-11. For this purpose, we conducted whole brain functional connectivity analyses in a data-driven way, with no use of predetermined seeds, using nonparametric tests to find significantly altered clusters of node links. Focus was given to the ASD and ADHD, as the primary comorbid conditions of interest, since these two neurodevelopmental disorders coexisted mostly in the GD patients who participated in the present study. In view of the behavioral association of ASD trait in problematic internet users [10,11], we expected that the ASD may have a substantial neurobiological impact on GD patients.

## Materials and methods

### Participants

The experiment was conducted with the approval of The Ethics Committee of Kurihama Medical and Addiction Center (KMAC), Human Bioethics Review Committee of Tokyo Denki University, and Ethics Review Committee on Research with Human Subjects of Waseda University. The aim of the study and procedures of MRI recording were explained to all participants and accompanying guardians of non-adult participants. Written form of informed consent was obtained from all prior to the commencement of experiment.

The patients participated in this study were outpatients of KMAC ranging from 12–26 years of age. They were diagnosed with GD by trained psychiatrists according to the criteria defined in the ICD-11 [1], which states that “in order for a diagnosis of the GD to be assigned, the gaming behavior and other features are normally evident over a period of at least 12 months, although the required duration may be shortened if all diagnostic requirements (briefly described at the top of Introduction) are met and symptoms are severe.” Here, the diagnosis of GD is not based on numerical measures. For all the patients, the diagnoses of ASD and ADHD were also conducted based on the criteria defined in Diagnostic and Statistical Manual of Mental Disorders, Fifth Edition (DSM-5) [48]. The ADHD section of the Japanese version of the Semi-Structured Interview for the Genetics of Alcoholism (version2) was also used to confirm the diagnosis of ADHD [49]. In addition, GD patients were diagnosed with disorders of depression, schizophrenia and other disorders using various instruments including the Mini International Neuropsychiatric Interview (MINI) [50]. For any conditions of ASD, ADHD, or other disorders, numerical measures on the severity of syndromes were not acquired or used as criteria of the diagnosis. Finally, the patients were separated into the GD individuals with no comorbid conditions (hereafter denoted by GD group) and those having comorbid conditions of ASD, ADHD, depression, or schizophrenia (denoted by GDcm group).

For healthy controls (HC), we recruited payed volunteers of young (17-year-old and below) and adult (above 17-year-old) groups from Junior-High/High Schools and Universities, respectively. Selection of the HC group was done by excluding individuals having GD, ASD, ADHD or other conditions in the following procedures. The young volunteers were given the same interviews, with accompanying parents/family, as the GD patients by the members of KMAC at the students’ school-campus. Based on these interviews, individuals who were suspected to have possibilities of GD and/or aforementioned conditions were screened out. For adult HC, volunteers of university students were administered a series of self-report questionnaires (Japanese-versions) of Internet Addiction Test (IAT) [51], Beck Depression Inventory-II (BDI-II) [52], Autism-Spectrum Quotient [53] and Adult ADHD Self Report Scale (ASRS -v1.1) [54]. The scores of these tests were used to find suspected individuals, adopting threshold scores of the questionnaires indicated in related literatures. The suspected individuals having scores equal or higher than any of these thresholds were excluded from the participants. Though not used in screening, the questionnaire of Barratt impulsiveness scale 11 was also administered [55]. After these tests, the selected adult HC individuals had mean scores of: IAT = 28.8 (40), BDI-II = 4.9 (11), AQ = 13.1 (34), ASRS = 1.6 (4) and BIS-11 = 57.1, where the adopted threshold scores are indicated in the parenthesis. The entire HC group comprised the selected young/adult participants.

Table 1 summarizes the information of the participants of the HC (n = 29), GD (n = 23) and GDcm (n = 17) groups whose data were analyzed in this study. Note, we removed two individuals (HC and GD) from the aforementioned selected participants based on a criterion of head motion during MRI recording, which was evaluated in the data preprocessing (described in Methods). The sex of all participants was male. The GDcm group comprised eight ASD, five ADHD, two ASD/ADHD, and two other (depression and schizophrenia) individuals. Analysis of variance (ANOVA) and post hoc tests revealed a difference in the mean age (p = 0.019) between the HC (19.4 years) and GD (17.0 years) groups. To compensate for this statistically significant age difference in the assessment of functional connectivity (FC) values while utilizing the data of all participants, we used weighted averaging of variables, a procedure which is described later. When we applied this method for the age assessment, nonsignificant difference (p = 0.118) was confirmed between the HC and GD groups. Between the GD and GDcm groups, there were no significant differences in the mean age, education period and gaming time/period. It is thus expected that the difference in the functional connectivity between the GD and GDcm groups would reflect effects of comorbidity conditions, under equal demographic characteristics.

**Table 1:** Demographic information and characteristics of participants.

	HC	GD	GD_cm_
Group size	29	23	17
Mean age (years)	19.4 ± 3.57	17.0 ± 3.50	17.2 ± 4.20
Education period (years)	12.1 ± 3.26	9.91±3.22	9.59 ± 3.05
Gaming time (hours/day)	1.52 ± 1.24	6.00 ± 2.78	6.13 ± 4.55
Gaming period (years)	N/A	9.57 ± 4.74	8.77 ± 5.95

HC: healthy control, GD: gaming disorder, GD_cm_: gaming disorder with comorbid conditions of ASD, ADHD, depression and schizophrenia.

### Recordings and data preprocessing

Recordings of functional magnetic resonance imaging were performed using a 3.0 T scanner (GE; Discovery MR 750w). Time signals of rs-fMRI were acquired in an echoplanar imaging (EPI) scheme with echo time (TE) = 30 ms, repetition time (TR) = 2800 ms and flip angle (FA) = 90°. The acquisition matrix was 64 × 64 in a field of view (FOV) of 240 mm. The slice thickness was 5 mm without gap, and 150 volumes of 28 slices were acquired in each participant. The participants were instructed to lie quietly, i.e., motionless, in the scanner with their eyes closed, not to sleep and, not to think about any events. Though no device was used for objective verification except for the scanner monitor, all participants subjectively reported that they were awake during the scan.

The first 10 volumes of the rs-fMRI signals were discarded to assure equilibrium of magnetization. Functional data of each participant were corrected for head movement, and a mean realigned image was created using the Statistical Parametric Mapping toolbox, version 8 (SPM8; Welcome Department of Cognitive Neurology, London, UK). Scan volumes exceeding 2-mm head movements were discarded. The data set was spatially normalized into the standard space defined by the Montreal Neurological Institute (MNI) template and spatially smoothed using 8-mm isotropic Gaussian kernel to compensate for inter-individual anatomical variance.

The framewise displacement of head motion in the rs-fMRI recording is a crucial index of data quality that may influence the connectivity network [56]. A criterion of the mean framewise displacement across volume scans (mFD) of 0.2 mm has been proposed, in a comprehensive comparative study [57], for the reduction of motion-related artifacts. Calculations of the mFD using a toolbox [58] revealed that two participants had excessive mFD values: 0.25 mm (2.6 times the mean of participant’s HC group); and 0.40 mm (3.2 times the mean of participant’s GD group). After removing these two participants, low mFD values of 0.09 ± 0.02, 0.11 ± 0.04 and 0.09 ± 0.03 mm were obtained for the HC, GD and GDcm groups, respectively. Though not significant, there was a marginal difference of the mFD among the three groups (p = 0.053, main effect of ANOVA). We did not implement the global signal regression for denoising as it might cause artifactual negative correlations [59, 60] or artifactual group differences in FC [61, 62], though the global signal has been regressed in some whole-brain FC studies for IA subjects [46, 47].

### Evaluation of functional connectivity

Time series of rs-fMRI signals were filtered to a frequency width of 0.017–0.09 Hz to extract blood-oxygen-level dependent (BOLD) signals. We analyzed the value of FC of the BOLD signals between anatomical areas of the brain defined by Automated Anatomical Labeling (AAL) [63], which parcels the brain into 116 areas. The AAL has commonly been used as an anatomical atlas in functional network studies [64], especially in whole brain functional connectivity analyses employing network-based statistics and topological theory [45–47, 65–67]. The cerebellum and vermis are divided into 30 areas in the AAL, which have many inter-areal connections (2665) between the cerebellum, vermis and other brain areas. We focused our functional connectivity analysis in the cerebral cortex and related regions such as the limbic and subcortical structures, by excluding connections with the cerebellum/vermis. Thus, we studied total 4005 connections across 90 AAL areas in the brain.

The functional connectivity is based on the correlation of time-series BOLD signals between a pair of nodes, which are given by AAL areas in this study. To calculate this correlation, time-series signals of all voxels in each AAL area were averaged to form a single waveform, and then Pearson’s correlation coefficient was calculated and used as the value of FC. In the assessment of FC for the HC and GD groups between which a small age difference of HC > GD existed, we adopted a method of weighted averaging to suppress the influence of the age difference. In this method, weighted group-mean FC is expressed by $\bar{FC}=\Sigma a_{i}FC_{i}∕\Sigma a_{i}$, where *a_i_* is the weight coefficient of participant *i*. The higher and lower mean ages of the HC and GD groups were mainly caused by high age HC-participants at 22 and 23 years-old (n_age_ = 5 each) and low age GD-participants at 13 and 14 years-old (n_age_ = 4 each), where n_age_ is the number of participants in an age-bin. These n_age_ exceeded the mean number of participants per age-bin across the two groups of n_mean_ = 3 (nearest integer to the exact value of 2.6). Then, we let the coefficients *a_i_* of these high- and low-age participants be *a_i_* = n_mean_/n_age_ to reduce and equate the contribution from the excess n_age_ participants to what would be given by n_mean_ participants. The *a_i_* of other participants were 1, as in the conventional averaging. We did not raise the *a_i_* of participants having the n_age_ lower than n_mean_, because enhancing the contribution of small number of participants per age-bin would increase errors. In the weighted averaging of group-mean dispersion $(\bar{SD^{2})}$, which was required in the calculation of t-test between groups, $\bar{FC}\;and\;FC_{i}$ in the above equation were replaced with $\bar{SD^{2}}$ and ${\left(FC_{i}-\bar{FC}\right)}^{2}$, respectively.

Following the above-mentioned procedures, we obtained the group-mean FC and its dispersion using the data of all participants in a group. We then conducted statistical evaluation, i.e., connection (node link)-wise comparisons, of the group-mean FC for 4005 node links in the whole brain between HC and GD, GD and GDcm, and HC and GDcm groups. We used Welch’s two-sample t-test [68] in the FC-comparison as this method is thought reliable when two groups have unequal dispersions and unequal sample sizes.

### Analyses of node links and core nodes

A large number of significant node links (> 200) were observed in between-group comparisons at a p-level of <0.05 (two-tailed). To focus on major node links associated with functionally important networks, we restricted node links by raising the significance level to p < 0.02. The obtained node links at this p-value were appropriate (see Table 2) to search for networks in the HC versus GD, and GD versus GDcm group-comparisons. After this connection-wise assessment, clusters of suprathreshold node links were identified in which all node links shared a common node as a center of the cluster (hereafter referred to “core node”).

**Table 2:** Brain regions and AAL areas of core nodes observed for the three group-contrasts of FC.

	Contrast (links)		
	HC > GD (51)	GD > GD_cm_ (117)	HC > GD_cm_ (1316)
Brain regions	AAL areas of core nodes (number of node links)		
Frontal	r-OLF (10), SFG_orb_ (6)	−	r-SFG_dsl_ (52), l/r-IFG_orb_ (49/59)
Frontal	−	−	r-SFG_med_ (49)
Central	r-PreCG (5), r-PCL (12)	−	r-PCL (45)
Temporal	−	r-TP_stg_ (13), l/r-TP_mtg_ (48/25)	l/r-TP_stg_ (67/72), l/r-TP_mtg_ (68/55)
Temporal		−	l/r-HES (50/53), r-ITG (61)
Parietal	−	−	r-IPL (58), l-ANG (39)
Occipital	r-CAL (5)	−	l-CAL (49), r-CUN (45)
Limbic	r- HIP (9)	−	r-PHG (46), r-MCC (55)

l/r = right/left hemisphere. Full names of abbreviated AAL areas are shown in the captions of Fig 2.

We evaluated the statistical significance of core nodes, i.e., cluster-level significance, using nonparametric permutation test that can control over Family-Wise Type 1 Error (FWE) in multiple comparisons in the whole brain. To develop a data-driven approach that does not rely on a priori concepts, we adopted the procedures described in previous literatures [65,69,70]. First, all participants in two groups to be compared were reassigned randomly to one of the two groups while keeping the sample number in each group unchanged. Appropriate weighted-averaging and Welch’s t-test were conducted for suprathreshold node links, from which a series of core nodes having a node link cluster were identified. Among those, the largest core node and its cluster size were recorded. We conducted 5000 repetitions of this process while permuting participants’ group assignments and determining the maximum cluster size. The results yielded a relationship between the maximum cluster-size and its occurrence probability. Finally, clusters with their size exceeding the maximum cluster-size at a given occurrence probability (threshold) were regarded as significant. The p-value of 0.02 in the initial connection-wise test was used as this threshold. We thereby obtained the core nodes having cluster-level significant node links for three between-group comparisons among the HC, GD and GDcm groups. Finally, we estimated networks consisting of significant node links to the core nodes; a network was composed by selecting AAL areas, known as network components, from counter nodes linking to core nodes.

To evaluate the significance of comorbidity conditions in the GD > GDcm group-contrast, we conducted connection-wise regression analyses of FC using three factors (regressors) of comorbidity, i.e., ASD, ADHD, and depression/schizophrenia for the significant node links that belonged to core nodes. The significance of the comorbidity of ASD or ADHD was determined by the p-value (< 0.05) of the regression coefficient.

## Results

### Group comparisons of functional connectivity

In between-group comparisons of FC at a significance level of p < 0.02, we obtained 51, 171 and 1316 connection-wise significant node links having HC > GD, GD > GDcm and HC > GDcm contrasts, respectively. Node links having opposite group contrasts were not observed at the p-level up to < 0.05. Table 2 shows brain regions and AAL areas of significant core nodes assessed by permutation tests, with cluster size (the number of connected nodes) shown in parentheses. Although the number of core nodes was fewer for the GD > GDcm compared to the HC > GD contrast (2 vs. 6), total node links were much greater for the former contrast because of greater cluster size. It was also found that the core node areas did not overlap between these two group-contrasts. These results imply that the spatial distribution of the node links converging to core nodes, in association with comorbid conditions, in the GD > GDcm contrast was distinct from the distribution of the node links in the HC > GD contrast, and that effects of comorbidities dominated the effects of gaming disorder.

**Fig 1:**
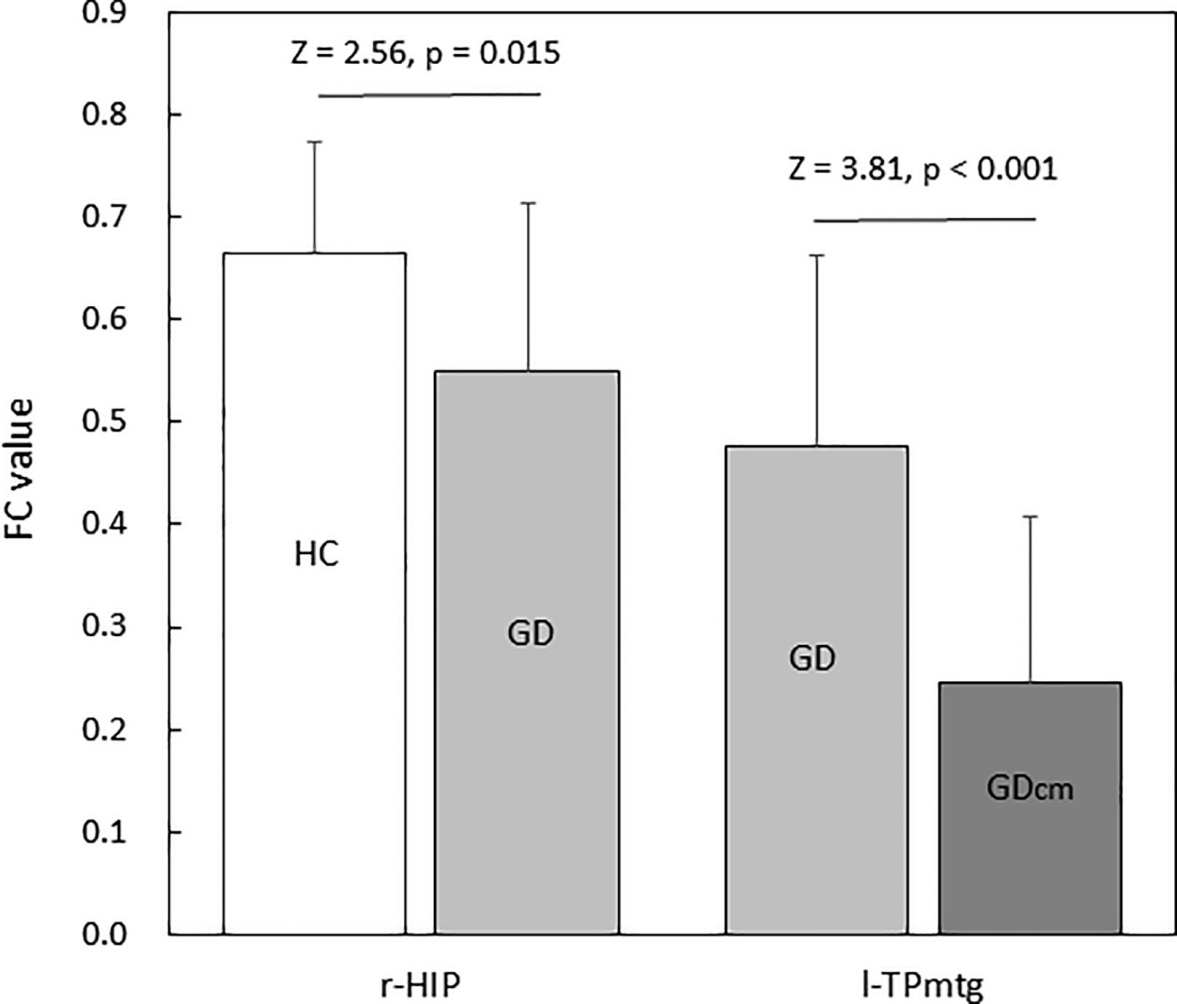
Mean FC-values for the principal core nodes of right hippocampus (r-HIP) in the HC versus GD group-comparison and left temporal pole (l-TPmtg) of the middle temporal gyrus in the GD versus GDcm group-comparison. Mean FC was calculated across the 10 lowest-p node links to the core node.

To quantitatively examine the alteration of connectivity, we calculated the group-mean FC-value of principal core nodes: right hippocampus (r-HIP) for the HC > GD group-contrast; and left temporal pole of middle temporal gyrus (l-TPmtg) for the GD > GDcm group-contrast. The results (Fig 1) indicated that the reduction of the mean FC, represented by the Z- and p- values, was obviously greater between GD and GDcm groups than between HC and GD groups. This is in line with the increase in cluster size of the core nodes and the total node links in the GD > GDcm group-contrast compared to the HC > GD group-contrast.

Although we set the significance level of node links and core nodes at p < 0.02 in this study, we made comparison of features of core nodes and functionally important networks with those obtained by applying a conventional threshold at p < 0.05. In the Supporting Information, S1 Table shows brain region, area and the number of significantly diminished node links observed at p < 0.02 and p < 0.05, for the FC contrasts of HC > GD and GD > GDcm groups. It was confirmed that core nodes at the lower threshold (p < 0.05) included all the core nodes and diminished links (k) at p < 0.02. Additional core nodes at p < 0.05 were found in the AAL areas contralateral to the core-node areas at p < 0.02, or mostly in the same brain regions of central, limbic and temporal areas. No overlapping of core-node areas between the two group-contrasts observed in this study (Table 2 in the text) was also maintained at p < 0.05. As seen in S2 Table, diminished core-node links that belong to the reward system (RWS) and networks (ECN and DMN) shared counter subregions of the brain at p < 0.05 with increased node numbers and at p < 0.02. Importantly, the absence of diminished-link nodes (the anterior cingulate cortex (ACC) and the putamen/pallidum (PUT/PAL) of basal ganglia) at p < 0.02 in the RWS for the HC > GD group-contrast was consistent at the lower threshold (p < 0.05). These two diminished areas were concordantly found at the two p-levels for the GD > GDcm group-comparison. Thus, the general features of core nodes and diminished system/network areas are consistent between the threshold of the present study and the conventional p-value of 0.05.

### Node-link areas in the HC > GD group-contrast

**Fig 2:**
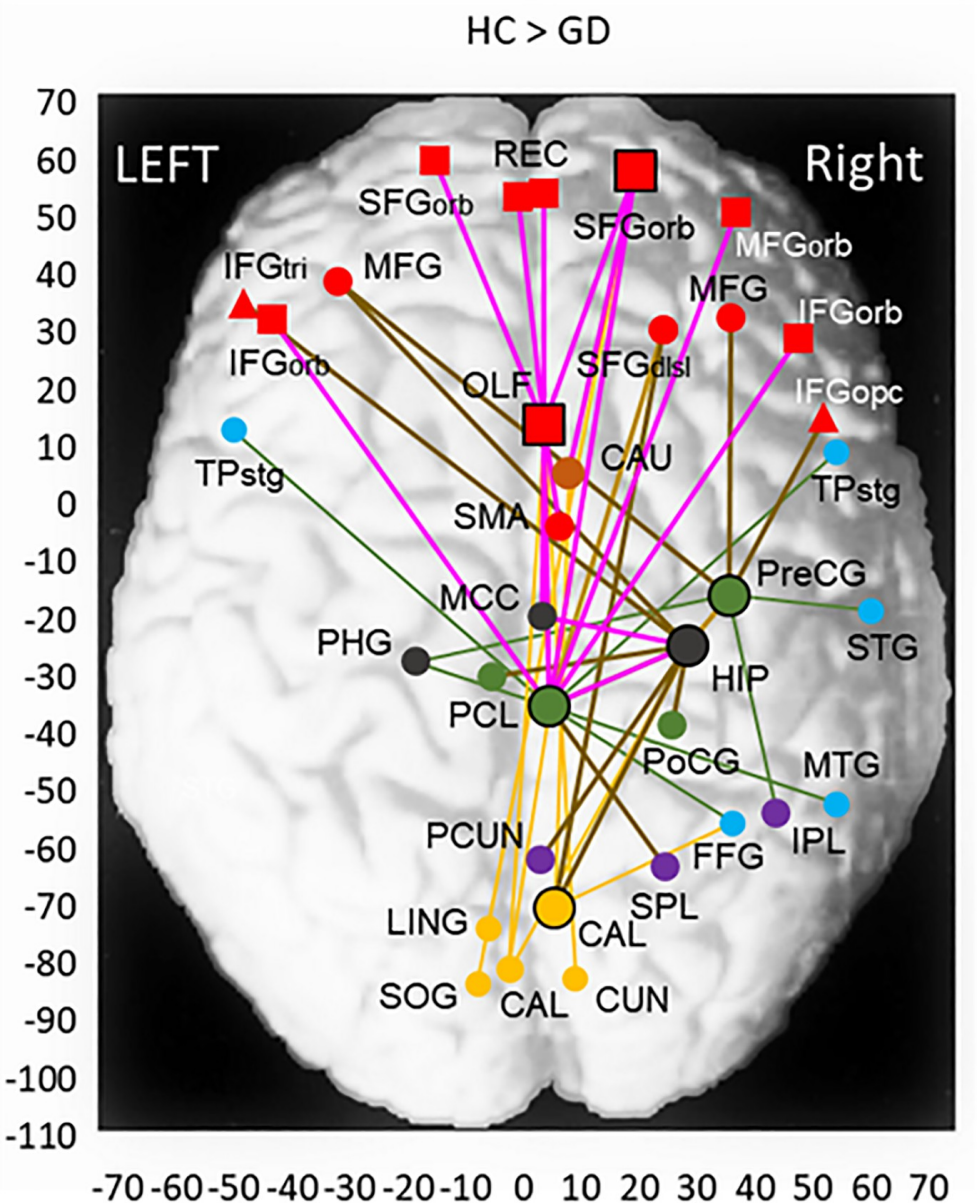
Two-dimensional view of core nodes (large symbols) and AAL node areas having a FC difference of HC > GD groups. MNI coordinates of core node areas are the maximum location of the mean BOLD signal in the HC group. The color of nodes indicates brain regions: red = frontal; green = central; blue = temporal; purple = parietal; orange = occipital; black = limbic, orange-brown = basal ganglia. The symbol of nodes indicates depths of node in the z-axis: square = orbital; triangle = ventral; circle = dorsal locations. Estimated network/system are indicated by color lines: dark brown = ECN; pink = RWS. Full names of abbreviated AAL areas used in the tables and figures: SFG/MFG/IFG = superior/middle/inferior frontal gyrus, REC = gyrus rectus, OLF = olfactory cortex, SMA = supplementary motor area, Pre/PoCG = pre/post central gyrus, PCL = paracentral lobule, STG/MTG/ITG = superior/middle/inferior temporal gyrus, TPstg/mtg = temporal pole of STG/MTG, HES = Heschl gyrus, FFG = fusiform gyrus, SMG = supramarginal gyrus, SPL/IPL = superior/inferior parietal lobule, PCUN = precuneus, ANG = angular gyrus, CAL = calcarine fissure and surrounding cortex, CUN = cuneus, LING = lingual gyrus, SOG/MOG/IOG = superior/middle/inferior occipital gyrus, ACC/MCC/PCC = anterior/middle/posterior cingulate cortex, AMG = amygdala, HIP/PHG = hippocampus/parahippocampal gyrus, INS = insula, CAU = caudate, PUT = putamen, PAL = globus pallidum, THL = thalamus. Portions of AAL areas: dsl/orb/med = dorsolateral/orbital/medial part, opc/tri = opercular/triangular part.

Node links to core nodes with significantly reduced FC were distributed in wide areas in the brain. We classified the brain areas of the counter node of node links into appropriate brain regions to estimate a network/system. It was assumed that a set of counter node areas in separate brain regions that were interconnected through a core node could form a network. Table 3 shows the core nodes identified for the HC > GD group-contrast with (x, y, z) MNI coordinates and the p-value of the core-node cluster, i.e., average p-value across node links. The number of node links to the core node is indicated in the row for each brain region. We selected appropriate brain regions of node links and assigned them to a plausible network based on a priori knowledge. The ECN and reward system (RWS) were estimated. Note that the frontal and parietal cortices were parceled into subregions consisting of one to four AAL areas so that each subregion corresponded to a separate component of a network/system. Fig 2 shows the spatial distribution of the core nodes and connected nodes with significantly reduced FC plotted on a horizontal (x, y) plane of MNI coordinates and superimposed on a schematic brain surface. The brain region is differentiated by the symbol color, and the color of connection lines indicates the estimated network/system.

**Table 3:** Cluster-level significant core nodes observed for the FC difference of HC > GD groups, and brain regions with the number of node links connected to the core node.

Core node	r-SFGorb	r-OLF	r-PCL	r-PreCG	r-CAL	r-HIP	
MNI coordinates	(19, 58, -13)	(3, 14, -14)	(4, -35, 76)	(35, -16, 64)	(5, -70, 11)	(28, -4, -5)	
Cluster-p	0.014	0.016	0.013	0.016	0.013	0.014	
Brain regions	Number of node links						*NW/SYS
Dorsolateral frontal		1		2	1	1	ECN (4)
Ventrolateral frontal				1		1	ECN (2)
Orbitofrontal	1	2	5				RWS (6)
Medial-orbitofrontal		2					RWS (2)
Central	1	2				3	(4)
Temporal			4	1	1		(5)
Inferior parietal				1			(1)
Posterior parietal			1		1	1	ECN (2)
Occipital	3	2				2	(6)
Middle cingulate		1				1	(1)
Hippocampus/PHG			2	1	1		RWS (2)
Caudate	1				1		(1)

*ECN = executive control network, RWS = reward system. The sum of nodes, not node links, in each brain region is indicated in the parenthesis of NW/SYS. AAL areas included in the brain regions related to the network/system are as follows: Dorsolateral frontal (SFGdsl/MFG); Ventrolateral frontal (IFGopc/tri); Orbitofrontal (SFGorb/MFGorb/IFGorb); Medial-orbitofrontal (REC); Inferior parietal (IPL); posterior parietal (SPL/PCUN/ANG). For the full name of abbreviated AAL areas, see captions of Fig 2.

The central executive network was originally proposed to consist of dorsolateral frontal cortex and posterior parietal cortex [71,72]. It was extended to the ECN by adding the ventrolateral frontal cortex, which may play a role in response suppression/inhibition and thus implements the control in executive function to operate as “executive control” [73,74]. In the estimated ECN shown in Fig 2, five significant nodes-link areas existed in the prefrontal cortex: three (r/l-MFG, r-SFGdsl) in the dorsolateral region, and two (l-IFGtri, r-IFGopc) in the ventrolateral region, where l/r indicates left/right hemispheres. Since the prefrontal cortex includes eight AAL areas of the ECN, it follows that five of eight (5/8) prefrontal ECN areas were occupied by significantly reduced-FC node links. In the posterior parietal region that consists of six AAL areas of the ECN, two significant nodes (2/6) existed in the r-PCUN and r-SPL areas. These node-link areas can briefly be seen in Fig 2. The prefrontal nodes were linked to core nodes of the precentral gyrus (r-PreCG) and hippocampus (r-HIP), to which the posterior parietal nodes were linked directly or via the core node of the paracentral lobule (r-PCL) core node. Thus, the dorso/ventrolateral frontal areas and the posterior parietal areas jointly formed an ECN having significantly reduced connectivities.

The RWS is based on the nucleus accumbens in the ventral striatum and limbic structures of amygdala and hippocampus [13], extending to the anterior cingulate cortex and the orbitofrontal cortex [14]. Among a total of ten areas in the orbitofrontal and medial orbitofrontal regions of the RWS, eight areas (8/10) were occupied by the significant node links, including the superior/medial/inferior frontal gyrus of orbital part (l/r-SFGorb/l-MFGorb/r-IFGorb), gyrus rectus (l/r-REC) and olfactory cortex (l/r-OLF) (Fig 2). Some of these nodes were linked to the core node of r-HIP through the r-OLF and middle cingulate cortex (r-MCC). Other orbitofrontal nodes were linked to a core node of the paracentral lobule (r-PCL), which had a link to the HIP core node. Thus, it seems that the HIP played a role as a center of the orbitofrontal connections in the RWS with significantly reduced FC. However, this RWS circuitry did not have components of the putamen (PUT) and globus pallidum (PAL) of the basal ganglia, which plausibly correspond to the ventral striatum. Thus, most of the orbital frontal areas were involved in the RWS, but the system was incomplete as crucial areas of the ventral striatum were absent.

### Node-link areas in the GD > GDcm group-contrast

**Fig 3:**
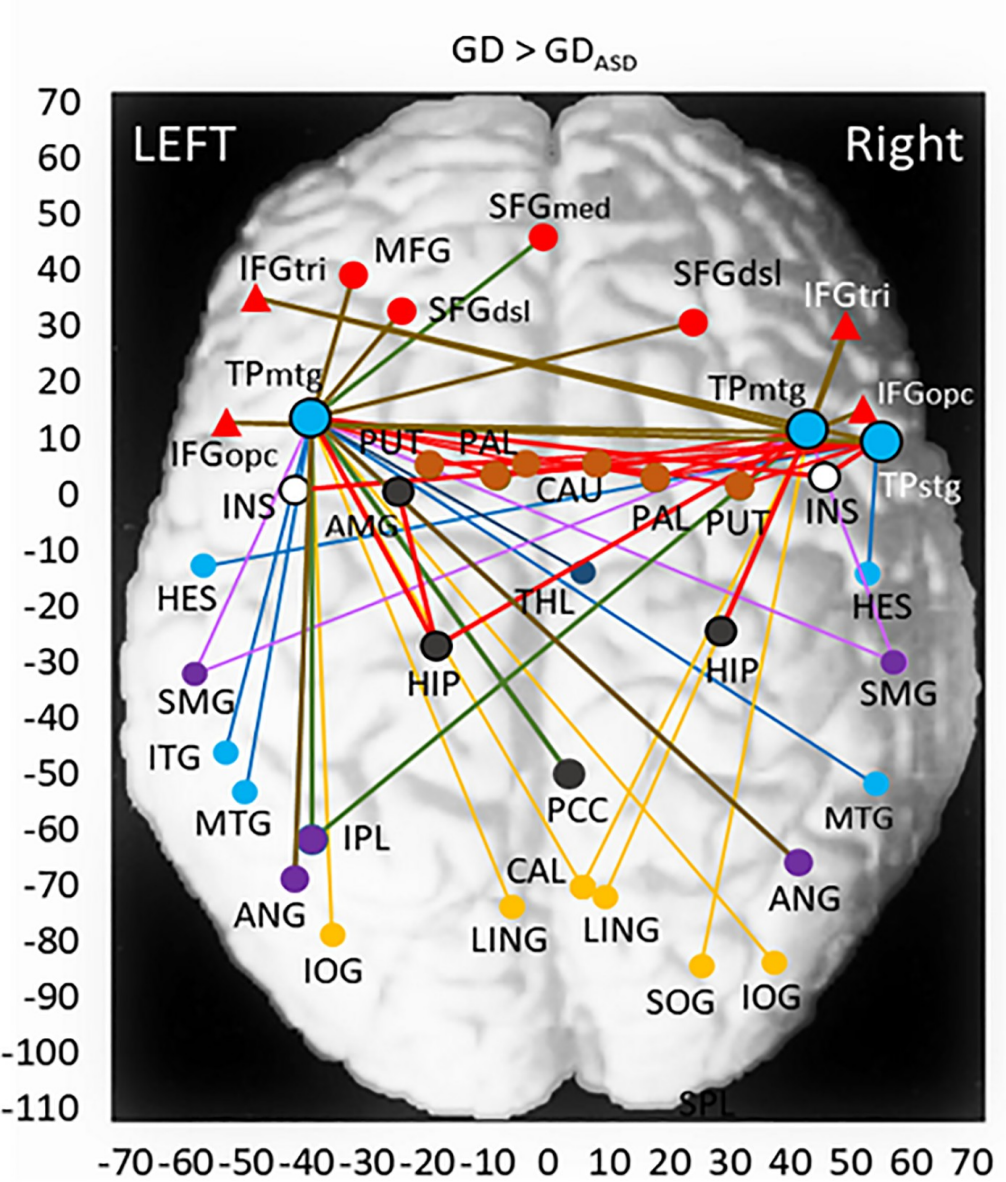
Two-dimensional view of core nodes (large symbols) and AAL node-link areas having a FC difference of GD > GDcm groups. Results are shown for the nodes significant to the ASD comorbid condition of the GDcm group. Symbols and lines are indicated as in Fig 2. The estimated DMN is indicated by green lines. Full names of abbreviated AAL areas are shown in the captions of Fig 2.

In the GD > GDcm group-contrast, 86 links to core nodes having significantly reduced FC were observed. They belonged to the core nodes of temporal poles of bilateral middle temporal gyri (l/r-TPmtg) and right superior temporal gyrus (r-TPstg). Using these data, 51 and 11 node links were determined as significant to the ASD- and ADHD-conditions with regression analysis, respectively. Table 4 shows the core nodes and brain regions that included node links to the core nodes, separately for the ASD and ADHD conditions. The spatial distribution of the core nodes and node link-areas plotted on the horizontal (x, y) plane of MNI coordinates are shown in Fig 3 for the ASD-significant condition.

**Table 4:** Cluster-level significant core nodes observed for the FC difference of GD > GDcm groups, and brain regions with the number of node links to the core nodes which were significantly related to ASD and ADHD conditions.

Core node	l-TP_mtg_	r-TP_mtg_	r-TP_stg_			l-TP_mtg_	r-TP_mtg_	
MNI coordinates	(-40, 14, -31)	(42, 11, -32)	(53, 9, -10)			(-40, 14, -31)	(42, 11, -32)	
Cluster-p	0.007	0.012	0.011			0.007	0.012	
Brain regions	ASD-related node links			(*Node)	NW/SYS	ADHD-related node links		(*Node)
Medial frontal	1			(1)	DMN			
Dorsolateral frontal	3			(3)	ECN			
Ventrolateral frontal		4	2	(4)	ECN			
Orbitofrontal					RWS	2		(2)
Insula	1		2	(2)				
Posterior cingulate	1			(1)	DMN	2	1	(2)
Temporal	3		2	(5)				
Temporoparietal JCT	2	2		(2)				
Inferior parietal	1	1		(1)	DMN			
Posterior parietal	2			(2)	ECN	3	2	(3)
Occipital	4	3		(6)				
Hippocampus		2		(2)	RWS			
Caudate	2		1	(2)				
Putamen/pallidum	4	3	4	(4)	RWS			
Thalamus	1			(1)		1		(1)

*Node is the sum of nodes, not node links, in each brain region. DMN = default mode network.

In clear contrast to the HC > GD difference, all areas of the basal ganglia (caudate, putamen, globus pallidum: CAU/PUT/PAL) were observed to have reduced-FC links to the core nodes of bilateral temporal poles (l/r-TPmtg and l-TPstg) in the ASD-significant condition. Here, the major part of the PUT is known to be included in the ventral striatum of the RWS together with the nucleus accumbens (NAc) [75]. The central location of the PAL at the group-mean BOLD maximum in this study was close to the coordinates of NAc reported in the previous study [76]. Thus, the area of PUT/PAL plausibly corresponds to the ventral striatum of the RWS reflecting the activity of the NAc. It was also found that the left amygdala (AMG) had reduced-FC links (p < 0.02) to the temporal poles (l/r-TPmtg) via the left HIP core node. Contrary to the full node links in the basal ganglia areas, no significantly reduced node links were found in the orbitofrontal regions of the RWS. However, if we combine the results of diminished node-link areas obtained for the HC > GD and ASD-related GD > GDcm group-contrasts (Tables 3 and 4 and Figs 2 and 3), it is expected that 8/10 (eight of ten) orbitofrontal areas, 4/4 PUT/PAL areas, 3/4 HIP/PHG areas of the RWS would have significantly reduced connectivities in a group comparison between HC and GDcm; the comorbidity of ASD condition in the GDcm patients would facilitate the FC reduction such that the connections in most areas of the mesolimbic circuit of the RWS are diminished.

As for the estimated ECN, reduced-FC node links existed in four AAL areas (l/r-IFGtri/opc) in the ventrolateral frontal region and in two posterior parietal areas of angular gyrus (l/r-ANG) that were not observed in the HC > GD group-contrast. If we combine the results for the HC > GD and GD > GDcm group-contrasts, significantly reduced-FC node links would be observed in 8/ 8 frontal areas and 4/6 posterior parietal areas of the ECN for the group comparison between HC and GDcm. This estimation suggests that the GDcm group having ASD comorbidity may have diminished connections in most of the ECN areas, compared to the HC group.

The DMN was revealed in imaging studies as a set of regions that consistently showed greater activity during resting states than during cognitive tasks [77,78]. Main brain regions of the DM network were shown to include the posterior cingulate cortex (PCC), inferior parietal lobule (IPL) and medial frontal cortex [79]. In Fig 3 and Table 4, reduced-FC node links existed in the superior frontal gyrus of medial part (l-SFGmed), the r-PCC and the l-IPL, from which a configuration of the DMN including the frontal, cingulate and parietal regions was inferred. However, the reduced-FC links were incomplete as a network and limited to the left hemisphere.

In significantly reduced node links observed for the ADHD-condition, bilateral core nodes of the temporal pole (l/r-TPmtg) were confirmed. AAL areas of the counter nodes indicated the olfactory cortex, posterior cingulate cortex, thalamus, angular gyrus and precuneus.

### Node-link areas in the HC > GDcm group-contrast

To examine the aforementioned predictions about combined results of the HC > GD and GD > GDcm group-contrasts, we sought AAL areas and their connections, which belonged to the ECN, RWS and DMN, from large number of significantly diminished node links in the HC > GDcm group-contrast (Table 2). To do this, we assigned the left and right temporal poles (l/r-TPmtg/stg) as core nodes and selected the links of the core nodes that had counter node-areas included in the above networks/system. The obtained results showed that the counter node areas of the selected links formed the ECN, RWS of almost completed structures but an incomplete structure of the DMN, as summarized in Table 5. The ECN consisted of diminished nodes in all bilateral dorsolateral and ventrolateral frontal areas and bilateral posterior parietal areas (SPL, ANG and PCUN). The diminished nodes in the RWS comprised bilateral areas of the orbitofrontal cortices, anterior cingulate cortex (ACC), amygdala (AMG), HIP, and PUT/PAL. The diminished nodes in the DMN were bilateral but only connected to a core node of the left temporal pole (TPstg). These results for the HC > GDcm group-contrast are consistent with the prediction that the ASD comorbidity would have effects on GDcm patients to extend and complete diminished ECN and RWS.

**Table 5:** Brain areas of the network and system having diminished links to the selected core nodes of temporal poles, observed for the HC > GD_cm_ group-contrast.

ECN		RWS		DMN
l/r-TPmtg	l/r-TPstg	l/r-TPmtg	l/r-TPstg	l-TPstg
AAL areas connected to the core nodes				
l/r-SFGdsl	l/r-SFGdsl		l/r-SFGorb	l/r-SFGmed
l/r-MFG	l/r-MFG	*l/r-MFGorb	*l/r-MFGorb	
l/r-IFGtri	l/r-IFGtri	l/r-IFGorb	l/r-IFGorb	
l/r-IFGopc	l/r-IFGopc		l/r-ACC	l/r-IPL
l/r-SPL	l/r-SPL	l/r-AMG	l/r-AMG	l/r-PCUN
l/r-ANG	l/r-ANG	l/r-HIP	l/r-HIP	l-PCC
l/r-PCUN	l/r-PCUN	l/r-PUT/PAL	l/r-PUT/PAL	

*Connected via the OLF area. Full names of AAL areas are described in the captions of Fig 2.

## Discussion

### Executive control network and reward system

We estimated diminished ECN and RWS from the clusters of reduced-FC nodes. In the diminished ECN observed in the GD group compared to the HC group, dorsolateral/ventrolateral prefrontal nodes and posterior parietal nodes were identified. Those areas were interconnected with reduced-FC mainly via the HIP core node (Fig 2). Similar configuration of the ECN was confirmed in the ASD-significant GDcm group compared with the GD group, in which diminished nodes were extended to new areas in the dorsolateral/ventrolateral frontal and posterior parietal regions. (Fig 3). These results suggest that the ECN is a fundamental mechanism that is diminished by the effect of gaming disorder alone, and that the comorbidity of ASD tends to extend the diminishment of the ECN in the frontal and posterior parietal regions. Finally, the ECN was diminished in almost all component areas in the ASD-significant GDcm group compared to the HC group. Previous imaging studies on IGD individuals have revealed negative correlation of the performance of executive/cognitive control, evaluated with Stroop test, with the functional connectivity within the ECN [27], and with functional and effective connections between the ECN and salience networks [31]. The outcomes of these activation studies are in line with and support the observation of diminished ECN in the GD group in this study.

Diminished connectivities of the RWS were observed in the GD group compared to the HC group in wide areas of orbitofrontal and limbic HIP/PHG regions, but not the basal ganglia areas (PUT/PAL) crucial for the RWS (Fig 2). These PUT/PAL areas, and the AMG and bilateral HIP were found with diminished connectivities in the ASD-significant GDcm group compared with the GD group. Finally, the orbitofrontal cortex, anterior cingulate cortex, amygdala, PUT/PAL and HIP were confirmed with diminished connectivities in the GDcm versus HC group comparison (Table 5). We presume that the diminished connections of the orbitofrontal, HIP and PUT/PAL (ventral striatum) areas represent the mesocortical and mesolimbic circuits [13].

Alteration of the mesocortical/limbic reward system has previously been associated with behavioral addictions [20]. In relation to the IGD, several studies have reported altered activities of the striatum: reduced levels of dopamine expression and dopamine receptor availability [21,22]; decreased activation during inhibition and reward-anticipation tasks [23,25]; and reduced functional connectivities with the dorsal PUT and dorsal striatum/PAL [24,26]. All these studies suggest decremental alteration of the striatum activity, which is consistent with our observation of the diminished frontal-to-ventral striatum connectivities in the GDcm group. Furthermore, the wide brain areas of the diminished RWS found in this study are coincident with the reported pattern of altered activation in heroin-dependent individuals, including prefrontal cortices, limbic structures and basal ganglia structures [80]. Taken together, the diminishment of the connectivity in the brain areas of orbitofrontal cortex, ventral striatum and limbic structures observed in the GDcm group with coexisting ASD, in comparison to the HC group, suggests that the mesocortical circuits of the RWS were disordered in a similar circuitry as in behavioral addiction. We presume that the coexistence of the ASD condition had substantial detrimental effects on the RWS in gaming disorder patients.

### Basis of the influence of comorbid conditions

We inferred effects of coexisting conditions of ASD/ADHD from the difference of FC between the GD and GDcm, and between the HC and GDcm groups. The primary finding in the GDcm group is the increase in spatial extent of diminished ECN and RWS in the entire network regions. We sought conceivable mechanisms underlying these alterations in the neurobiological findings revealed by brain imaging.

Previous studies for autism patients and autistic individuals indicated: reduced regional cerebral blood flow in the prefrontal cortex/insula, and specific perfusion pattern in the medial prefrontal and limbic regions in children with infantile autism [81]; delayed maturation, i.e., hypoperfusion, of the frontal cortex at ages of 3–4 years and normal values by 6–7 years in autistic children [82]; low correlation of regional cerebral metabolic rates of glucose (functional interaction) between frontal/parietal and other cortical/subcortical regions in healthy autistic males [83]; and reduced activation during spatial working memory task in dorsolateral prefrontal cortex and posterior cingulate cortex in autistic subjects [84]. As for patients/individuals with ADHD, previous studies have shown reduced activation [85], reduced structural connectivity [86], correlation of the grey matter volume with the severity of symptoms [87] and decreased gray matter volume [88] in the prefrontal cortices and related structures. These studies suggest that young individuals with ASD and ADHD tend to have metabolic, structural and functional anomalies in the prefrontal cortex and its connections to other brain regions.

Provided that incompleteness exists in the function and/or structure of prefrontal cortex in the GDcm group with coexisting ASD/ADHD, this weakened prefrontal activity might facilitate the reduction of prefrontal-based connectivities in a network. In line with this, diminished node links were found in almost all AAL areas of dorsolateral/ventrolateral and orbital prefrontal regions of the ECN and RWS in the GDcm group compared to the HC group, as described so far.

### Core nodes

Core nodes were defined as the central node having a cluster of significantly diminished connections to other counter-node areas. The observation of the core nodes is an important outcome of cluster-based statistics, in which clusters of maximal size are searched [70,89]. In the HC > GD group-contrast of FC, the HIP core node had reduced-FC links to the primary visual areas (CAL) bilaterally (Fig 2), while other core nodes (SFGorb, OLF) had reduced-FC links to higher visual areas (LING, SOG, CUN) in the occipital cortex. These observations imply that the HIP node area specifically received visual information from the primary visual cortex through diminished connections.

With respect to the visual processing, a visual information pathway coursing from the parietal lobe downward to the HIP in the medial temporal lobe [90] is known in the dorsal visual-information streams. This pathway subserves spatial navigation, whereas traditional parieto-prefrontal and parieto-premotor pathways support spatial working memory and visually guided action, respectively. In line with this notion, the damage to the parieto-medial temporal pathway was reported to result in topographic disorientation in human [91]. Meanwhile, neurons in human entorhinal cortex, which is a main input area to the HIP, were activated during a virtual navigation task [92]. In addition, the HIP was involved in visual discrimination of complex spatial-scene stimuli [93]. The results of these studies indicate that the HIP engages in the spatial navigation and visual discrimination, which should be important in the gaming such as visually-guided-actions in varying background environments. This kind of visual perception and cognition may be additive to the main function of memory of the HIP associated with emotion and learning.

These versatile functions of the HIP suggest the possibility that the HIP plays a role as a central core node in the functional network/system, operating multiple-information processing. It should also be mentioned that the HIP/PHG have vulnerability to structural and neuronal changes by intensive game playing. Reported altered quantities include the grey matter volume of the PHG and occipital/parietal cortices [94], the grey matter density of the HIP, precuneus, inferior frontal gyrus and others [95], and the connectivity between the HIP/PHG and the fronto-executive system [96]. Such vulnerability may contribute to the diminishment of node links to the HIP core node.

An interesting finding is the emergence of the temporal pole core nodes (TPmtg/stg) in the GD versus GDcm group-comparison. Anatomical and functional connectivity studies have shown that the anterior temporal lobe has connections, in its parceled subregions, with wide brain regions including auditory, somatosensory, visual, and olfactory areas [97,98]. Thus, the anterior temporal lobe serves as a converging center of the posterior sensory information. It was proposed that the anterior temporal lobe acts as a binding hub for semantic memories of different sensorimotor modalities to form concepts [99]. As for pathways, information from the posterior sensory/association cortices is transferred to the anterior temporal lobe through nerve bundles of (a part of) superior, middle and inferior longitudinal fasciculi [100,101], some of which may overlap with the ventral visual-information pathway. Then, the converged information at the anterior temporal lobe is transferred to prefrontal cortices through the uncinate fasciculus. The importance of the uncinate fasciculus as a bridging pathway between the anterior temporal lobe and the orbitofrontal cortex has been pointed out in a review [102].

Taken together, the previous findings for the anterior temporal lobe suggest a possibility that the temporal-pole operate as a converging center of the information from posterior cortical cortices. This notion is supported by the node links from wide regions of the occipital, temporal and parietal cortices to the temporal-pole core nodes observed in the GD > GDcm group-contrast (Fig 3). We suppose that GD individuals, especially with ASD/ADHD comorbidities, utilize abundant information of visual, auditory, and somatosensory stimuli during gaming, and that this information is transferred via temporal poles and processed in the orbitofrontal cortices of the RWS. It should be noted that the anterior temporal lobe has close intrinsic connectivity with subcortical regions including the ventral striatum, AMG, and others, in addition to orbitofrontal cortices [103]. Therefore, it is plausible that the links of the PUT/PAL, ACC, AMG, and HIP share the core nodes of the temporal pole with the links of the orbitofrontal areas in the common RWS, and that these node links jointly have diminished connectivities in the GDcm individuals having comorbid conditions. This connection scheme of the node links and the diminishment of FC are consistent with the observations in the HC > GDcm group-contrast (Table 5).

### Methodological issues/limitations

Functional connectivities and network measures derived from rs-fMRI signals are susceptible to head motion [56,104]. Various approaches such as global signal regression, framewise-displacement regression and scrubbing have been proposed and evaluated to correct for the head motion artifacts, whereas they may further affect the FC estimates depending on the strategies to be used [56,57,105–107]. We adopted an approach of thresholding the mean framewise-displacement (mFD) [57] and obtained low mFD values for the HC, GD, and GDcm groups. It is expected that the variation of the FC with mFD within a group may not directly affect the between-group comparison of FC unless difference exists in the mean mFD. However, there was a marginal group difference of mFD (0.02 mm greater in the GD group than the HC and GDcm). Although greater head motion is thought to underestimate the FC [104], it is not clear whether the remained mFD difference affects the group comparison of FC. Furthermore, any difference of mean mFD between groups should be confounded with the factor of group in regression analysis. Thus, difficulty may exist to interpret the outcomes when the mFD is regressed out in the evaluation of the FC. How to reduce head motion artifacts precisely in the estimation of group difference of FC is a subject of future investigation.

A limitation is the small sample size of participant groups, especially of GDcm group. We conducted connection-wise regression analyses to select ASD- and ADHD-significant node links for the GD > GDcm group-contrast. Of 86 diminished-FC node links to the core nodes (TPmtg/stg), 51 nodes were significant to the ASD condition, but only 11 nodes were significant to the ADHD. Although this result suggests a minor impact of the ADHD relative to the ASD, reduced statistical power due to a small sample size of ADHD group (n = 7 including ASD/ADHD conditions) should have detrimental effect. Further research is required with greater population of patients to differentiate the network structure depending on the comorbidity of ASD and ADHD.

In summary, we investigated effects of comorbidity conditions on the gaming disorder. Substantial impact of the ASD was revealed, extending diminished areas of the ECN and RWS in the entire network regions. This finding suggests that the coexisting ASD facilitates impairment of executive/cognitive and emotional functions in the individuals with gaming disorder. We presume that the whole brain resting state FC (rsFC) analysis, without using predetermined seed regions, made it possible to estimate the ECN and RWS in a single investigation. To our knowledge, this is the first investigation reporting the influence of comorbidity of ASD on the rsFC in gaming disorder patients. The association of the diminished ECN/RWS, evaluated with the rsFC, with behavioral measures of GD/GDcm patients is a subject of future study.

## Supporting information

S1 Table Brain regions, AAL areas and the number of links (k) to core nodes observed at higher (p < 0.02) and lower (p < 0.05) significance levels. (DOCX)

S2 Table Subregions of the brain and the number of included nodes that belong to RWS, ECN and DMN. (DOCX)
