# Supplementary material for: Neurobiological influence of comorbid conditions in young patients diagnosed with gaming disorder: A whole-brain functional connectivity study based on a data driven method
Source: PLoS One. 2020 May 29;15(5):e0233780. doi: 10.1371/journal.pone.0233780 (PMC7259694; doi:10.1371/journal.pone.0233780)
Supplement: S1 Table — (DOCX) [file pone.0233780.s001.docx]

**Table S1**. **Brain regions, AAL areas and the number of links (k) to core nodes observed at higher (p < 0.02) and lower (p < 0.05) significance levels**. Underlines indicate the core nodes shown in Table 3. See captions in Fig. 2 for the abbreviated AAL areas.

| Regions | AAL areas | k (<0.02) | k (<0.05) |
| --- | --- | --- | --- |
| HC > GD group-contrast | | | |
| Frontal | r-SFGorb | 6 | 20 |
| Frontal | r-OLF | 10 | 20 |
| Central | r-PCL | 12 | 26 |
| Central | l-PCL | − | 12 |
| Central | r-PreCG | 5 | 21 |
| Central | r-PoCG | − | 16 |
| Occipital | r-CAL | 5 | 18 |
| Occipital | l-CAL | − | 14 |
| Limbic | r-HIP | 9 | 23 |
| Limbic | l-HIP | − | 15 |
| Limbic | r-PHG | − | 12 |
|  | **Sum** | **47** | **197** |
| GD > GDcm group-contrast | | | |
| Temporal | r-TPmtg | 25 | 42 |
| Temporal | l-TPmtg | 48 | 73 |
| Temporal | r-TPstg | 13 | 33 |
| Temporal | l-TPstg | − | 14 |
| Temporal | l-FFG | − | 24 |
| Temporal | r-ITG | − | 13 |
| Occipital | r-IOG | − | 16 |
|  | **Sum** | **86** | **215** |
