# Supplementary material for: Neurobiological influence of comorbid conditions in young patients diagnosed with gaming disorder: A whole-brain functional connectivity study based on a data driven method
Source: PLoS One. 2020 May 29;15(5):e0233780. doi: 10.1371/journal.pone.0233780 (PMC7259694; doi:10.1371/journal.pone.0233780)
Supplement: S2 Table — (DOCX) [file pone.0233780.s002.docx]

**Table S2. Subregions of the brain and the number of included nodes that belong to RWS, ECN and DMN.** The number of nodes was given from links to the core nodes in Table S1 having a reduced FC at p < 0.05, while the number of nodes in the parenthesis was from reduced-FC links at p < 0.02. Note, complete forms of the RWS, ECN, and DMN consist of total 22, 16 and 6 nodes, respectively, in different subregions across right and left hemispheres. The DMN had no significant core-node links for the HC > GD group-contrast. Details of the configuration of RWS, ECN and DMN are described later in corresponding sections.

| Subregions | RWS-nodes | ECN-nodes | DMN-nodes |
| --- | --- | --- | --- |
| HC > GD group-contrast | | | |
| Orb-FR | 10 (8) |  |  |
| ACC | ns (ns) |  |  |
| HIP/PHG | 3 (2) |  |  |
| PUT/PAL | ns (ns) |  |  |
| Dsl/vtr-FR |  | 9 (6) |  |
| Pos-PR |  | 5 (2) |  |
| **Sum** | **13 (10)** | **14 (8)** |  |
| GD > GDcm group-contrast | | | |
| Orb-FR | 11 (8) |  |  |
| ACC | 2 (1) |  |  |
| HIP/PHG | 4 (3) |  |  |
| PUT/PAL | 4 (4) |  |  |
| Dsl/vtr-FR |  | 10 (9) |  |
| Pos-PR |  | 6 (5) |  |
| Med-FR |  |  | 2 (1) |
| PCC |  |  | 2 (2) |
| Inf-PR |  |  | 2 (2) |
| **Sum** | **21 (16)** | **16 (14)** | **6 (5)** |

Subregions: orb/dsl/vtr/med-FR = orbital/dorsolateral/ventrolateral/medial-Frontal, pos/inf-PR = posterior/inferior-Parietal.
